# The pros and cons of degeneracy

**DOI:** 10.7554/eLife.02615

**Published:** 2014-04-01

**Authors:** Jean-Marc Goaillard, Martial A Dufour

**Affiliations:** 1 **Jean-Marc Goaillard** is at Inserm (Inserm UMR_S 1072) and Aix Marseille University, Marseille, Francejean-marc.goaillard@univ-amu.fr; 2 **Martial A Dufour** is at Inserm (Inserm UMR_S 1072) and Aix Marseille University, Marseille, France

**Keywords:** neuropathic pain, degeneracy, excitability, membrane potential oscillations, dynamic clamp, bursting, rat

## Abstract

Drugs could treat neuropathic pain more effectively if they simultaneously targeted two or more types of ion channel.

**Related research article** Ratté S, Zhu Y, Lee KY, Prescott SA. 2014. Criticality and degeneracy in injury-induced changes in primary afferent excitability and the implications for neuropathic pain. *eLife* **3**:e02370. doi: 10.7554/eLife.02370

**Image** Changes in the sodium (horizontal axis) and potassium (vertical) conductances modify the firing behaviour of neurons

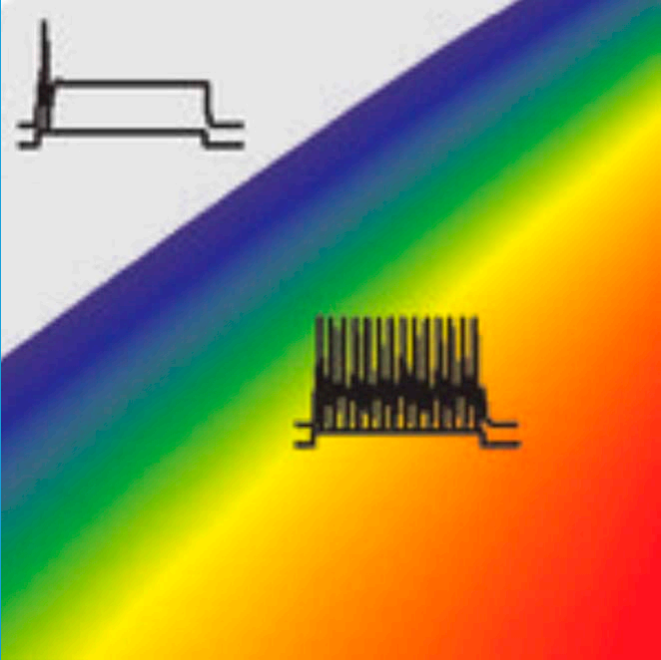

Complex biological systems are able to use multiple different solutions to produce similar phenotypes or outputs. This property is called degeneracy (Edelman and Gally, 2001) and it makes biological systems more robust because it reduces the impact caused by changes to a single parameter. In the genetic code, for instance, various combinations of three nucleotides can encode the same amino acid: for example, all nucleotide triplets starting with UC encode serine, which means that any mutation in the last nucleotide (which can be U, C, G or A) does not affect the protein that is produced (Goldberg and Wittes, 1966).

Early in the study of genetics, the embryologist Conrad Hal Waddington demonstrated that the development of biological organisms is protected from isolated genetic variations because numerous genes with overlapping functions define how development proceeds (Waddington, 1957). This suggested for the first time that there is a direct link between degeneracy and biological robustness. Now, in *eLife*, Steven Prescott and colleagues at the University of Toronto and the University of Pittsburgh—including Stéphanie Ratté as first author—show that degeneracy may also underlie the robustness of pathological states against pharmacological treatments (Ratté et al., 2014).

Neurons conduct information along their length through the firing of action potentials. These are electrical signals generated by the movement of ions through channels in the nerve membrane. Action potentials rely on the activity of many different subtypes of ion channels (including potassium, sodium and calcium channels, and also non-specific channels; Bean, 2007), which therefore define the excitability profile of neurons (which is the ability of neurons to generate an action potential in response to a given stimulus). Theoretical and experimental studies have demonstrated that neurons are able to generate the same excitability profile in many different ways (Marder and Goaillard, 2006; Marder and Taylor, 2011), to the extent that normal levels of excitability can be maintained even if a major ion channel is chronically deleted, as seen in knock-out animals.

Neuropathic pain occurs after lesions in the peripheral or central nervous system and involves the appearance of highly debilitating spontaneous pain and hypersensitivity to both painful stimuli (a form of pain known as hyperalgesia) and sensory stimuli (dysesthesia and allodynia; Costigan et al., 2009). Neuropathic pain is currently interpreted as a maladaptive plasticity response of the nervous system to injury. The causes and the pain phenotypes associated with the disease are complex, but it has been established that neuropathic pain results from changes in how the synapses between nerve cells work and how action potentials are generated. Instead of firing a single action potential in response to a stimulus, neurons fire many action potentials repetitively. This is known as neuronal hyperexcitability.

Ratté et al. studied how the degeneracy involved in generating action potentials may affect the development of the hyperexcitability associated with neuropathic pain. First, they showed that the change in neuron behaviour from normal physiological states to pathological states of excitability occurs when a tipping point is crossed (Figure 1). Using pharmacological blockade, electrophysiological recordings and computational modelling, Ratté et al. then demonstrated that this tipping point can be crossed by changes in the conductance of different ion channels. In naïve (non-injured) animals, hyperexcitability can be generated by an increase in sodium conductance, a decrease in potassium conductance, or both. Applying the opposite changes—decreasing the sodium conductance, increasing the potassium conductance, or both—can reverse the hyperexcitability observed in nerve-injured animals experiencing neuropathic pain. The discovery of these multiple ways of controlling the excitability reveals there is a substantial level of degeneracy behind the action potential response.

**Figure 1.**
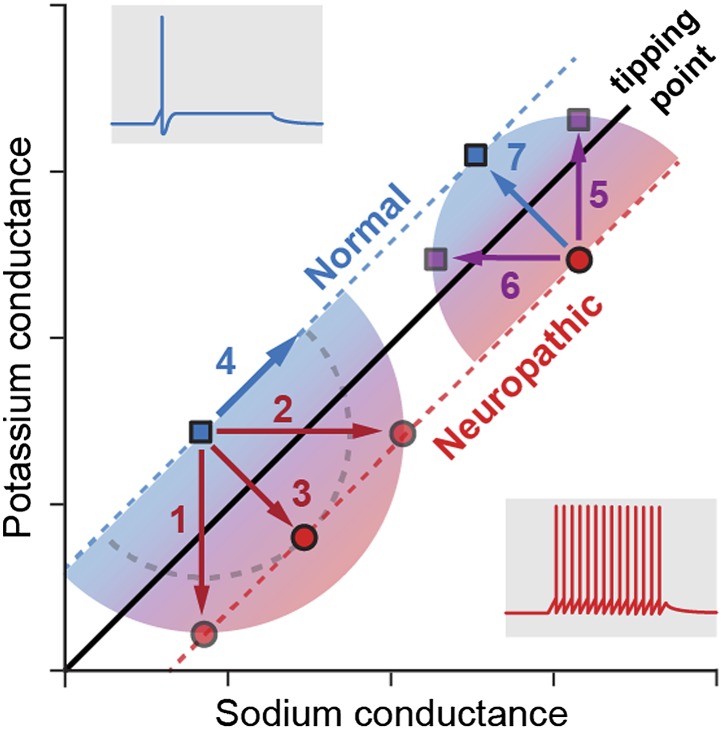
Multiple pathways to switch between normal excitability and hyperexcitability in a neuron. Primary afferent neurons can display a normal excitability profile, characterized by the firing of a single action potential (blue trace) in response to a current step, or a ‘neuropathic’ hyperexcitability profile, characterized by the repetitive firing of action potentials in response to the same stimulus (red trace). Ratté et al. have found that the switch between these two profiles occurs when a tipping point is crossed, and this transition can be induced by varying the sodium (horizontal axis) and potassium conductances (vertical). For instance (left part of the graph), decreasing the potassium conductance (1), increasing the sodium conductance (2) or applying both changes simultaneously (3) induces the transition from normal to neuropathic behaviour, while increasing both conductances (4) preserves a normal excitability profile. Conversely, reversing the neuropathic profile can be achieved by increasing the potassium conductance (5), decreasing the sodium conductance (6) or applying both changes simultaneously (7). Note that, for conductance modifications of the same magnitude (arrows 5, 6 and 7), concerted changes in both conductances are much more efficient in reversing the neuropathic phenotype: the purple squares reached with arrows 5 and 6 are much closer to the tipping point than the blue square reached with arrow 7. This may explain why drugs targeting single ion channels are less efficient at treating neuropathic pain.

This finding sheds light on the surprising results obtained in transgenic animals lacking two types of sodium ion channels called Nav1.7 and Nav1.8 (Nassar et al., 2005). While both of these sodium channels are highly expressed in the neurons responsible for responding to pain, and have been demonstrated to be involved in neuropathic pain (Dib-Hajj et al., 2010), genetically suppressing these two channels in the pain-transmitting nerves does not affect the development of neuropathic pain. In other words, these neurons preserve their distance from the tipping point, even in the absence of Nav1.7 and Nav1.8. Based on these results, one can imagine that the reason why animals with deleted Nav1.7 and Nav1.8 sodium channels still experience neuropathic pain is because the potassium conductance correspondingly decreases to match the decrease in sodium conductance. This therefore maintains the same excitability behaviour.

Ratté et al. also demonstrate that simultaneously increasing the sodium conductance and decreasing the potassium conductance is the most efficient way to trigger the hyperexcitability associated with neuropathic pain, whilst the opposite conductance changes are the most efficient way of relieving it (Figure 1). As a consequence, drugs targeting a single ion channel are likely to be far less efficient at treating neuropathic pain than drugs targeting two ion channels. This is highly reminiscent of the observation that epilepsy (another excitability disorder) seems to develop in response to the combined misregulation of different ion channels (Klassen et al., 2011). ‘Dirty’ drugs targeting several ion channels might therefore be more efficient in treating both epilepsy and neuropathic pain.

Altogether, the work of Ratté et al. demonstrates that excitability disorders such as neuropathic pain rely on the degeneracy of excitability, with numerous ion channels defining the excitability profile of the neuron. These results call for our principles of drug design for excitability disorders to be reconsidered, to take into account how the degeneracy of biological systems helps develop pathological robustness and resistance to pharmacology.
